# Supplementary material for: Modeling glioblastoma heterogeneity as a dynamic network of cell states
Source: Mol Syst Biol. 2021 Sep 16;17(9):e10105. doi: 10.15252/msb.202010105 (PMC8444284; doi:10.15252/msb.202010105)
Supplement: Supplementary file 5 — Source Data for Figure 3 [file MSB-17-e10105-s001.zip › Figure3A_sourcedata/GSEA_3065/hallmarks_state3.GseaPreranked.1623416274744/HALLMARK_PROTEIN_SECRETION.html]

Details for gene set HALLMARK\_PROTEIN\_SECRETION[GSEA]

|  || Dataset | state3 |
| Phenotype | NoPhenotypeAvailable |
| Upregulated in class | na\_pos |
| GeneSet | HALLMARK\_PROTEIN\_SECRETION |
| Enrichment Score (ES) | 0.37620547 |
| Normalized Enrichment Score (NES) | 1.4410278 |
| Nominal p-value | 0.013550135 |
| FDR q-value | 0.07959488 |
| FWER p-Value | 0.332 |
Table: GSEA Results Summary

  

Fig 1: Enrichment plot: HALLMARK\_PROTEIN\_SECRETION      
 Profile of the Running ES Score & Positions of GeneSet Members on the Rank Ordered List

  

| PROBE | GENE SYMBOL | GENE\_TITLE | RANK IN GENE LIST | RANK METRIC SCORE | RUNNING ES | CORE ENRICHMENT || 1 | LMAN1 |  |  | 21 | 0.496 | 0.0412 | Yes |
| 2 | CD63 |  |  | 26 | 0.480 | 0.0828 | Yes |
| 3 | DST |  |  | 80 | 0.365 | 0.1088 | Yes |
| 4 | LAMP2 |  |  | 154 | 0.309 | 0.1275 | Yes |
| 5 | CLTC |  |  | 177 | 0.296 | 0.1510 | Yes |
| 6 | YIPF6 |  |  | 200 | 0.283 | 0.1733 | Yes |
| 7 | CTSC |  |  | 226 | 0.272 | 0.1943 | Yes |
| 8 | CLCN3 |  |  | 251 | 0.263 | 0.2146 | Yes |
| 9 | ATP1A1 |  |  | 285 | 0.249 | 0.2327 | Yes |
| 10 | RER1 |  |  | 294 | 0.244 | 0.2532 | Yes |
| 11 | IGF2R |  |  | 348 | 0.230 | 0.2673 | Yes |
| 12 | COPB2 |  |  | 353 | 0.229 | 0.2869 | Yes |
| 13 | TMED10 |  |  | 378 | 0.223 | 0.3038 | Yes |
| 14 | COPB1 |  |  | 405 | 0.218 | 0.3199 | Yes |
| 15 | M6PR |  |  | 520 | 0.196 | 0.3240 | Yes |
| 16 | PPT1 |  |  | 657 | 0.177 | 0.3238 | Yes |
| 17 | ERGIC3 |  |  | 704 | 0.169 | 0.3334 | Yes |
| 18 | KIF1B |  |  | 767 | 0.161 | 0.3404 | Yes |
| 19 | USO1 |  |  | 810 | 0.157 | 0.3493 | Yes |
| 20 | ARFGEF1 |  |  | 815 | 0.156 | 0.3625 | Yes |
| 21 | GLA |  |  | 816 | 0.156 | 0.3762 | Yes |
| 22 | CLN5 |  |  | 934 | 0.145 | 0.3754 | No |
| 23 | TMX1 |  |  | 1053 | 0.134 | 0.3736 | No |
| 24 | AP2B1 |  |  | 1478 | 0.105 | 0.3338 | No |
| 25 | VPS45 |  |  | 1507 | 0.103 | 0.3396 | No |
| 26 | ANP32E |  |  | 1548 | 0.101 | 0.3438 | No |
| 27 | AP1G1 |  |  | 1679 | 0.093 | 0.3370 | No |
| 28 | MON2 |  |  | 1815 | 0.086 | 0.3289 | No |
| 29 | SCAMP1 |  |  | 1911 | 0.080 | 0.3250 | No |
| 30 | GOLGA4 |  |  | 2023 | 0.075 | 0.3188 | No |
| 31 | TMED2 |  |  | 2244 | 0.066 | 0.2992 | No |
| 32 | ATP6V1H |  |  | 2249 | 0.066 | 0.3046 | No |
| 33 | ABCA1 |  |  | 2275 | 0.065 | 0.3074 | No |
| 34 | COG2 |  |  | 2308 | 0.064 | 0.3094 | No |
| 35 | ADAM10 |  |  | 2347 | 0.063 | 0.3105 | No |
| 36 | EGFR |  |  | 2416 | 0.060 | 0.3079 | No |
| 37 | GBF1 |  |  | 2435 | 0.059 | 0.3111 | No |
| 38 | SEC24D |  |  | 2587 | 0.054 | 0.2984 | No |
| 39 | ZW10 |  |  | 2625 | 0.052 | 0.2987 | No |
| 40 | STAM |  |  | 2650 | 0.051 | 0.3005 | No |
| 41 | AP3B1 |  |  | 2869 | 0.045 | 0.2793 | No |
| 42 | DNM1L |  |  | 2983 | 0.041 | 0.2699 | No |
| 43 | STX16 |  |  | 3016 | 0.041 | 0.2697 | No |
| 44 | SCRN1 |  |  | 3507 | 0.028 | 0.2156 | No |
| 45 | PAM |  |  | 3683 | 0.023 | 0.1975 | No |
| 46 | ARFGEF2 |  |  | 3736 | 0.022 | 0.1934 | No |
| 47 | ARFIP1 |  |  | 3741 | 0.022 | 0.1948 | No |
| 48 | ARCN1 |  |  | 3893 | 0.018 | 0.1790 | No |
| 49 | STX12 |  |  | 4016 | 0.015 | 0.1662 | No |
| 50 | VAMP4 |  |  | 4212 | 0.009 | 0.1445 | No |
| 51 | ARFGAP3 |  |  | 4451 | 0.002 | 0.1172 | No |
| 52 | SEC22B |  |  | 4515 | 0.001 | 0.1100 | No |
| 53 | SSPN |  |  | 4525 | 0.001 | 0.1090 | No |
| 54 | SCAMP3 |  |  | 4651 | -0.002 | 0.0948 | No |
| 55 | OCRL |  |  | 4913 | -0.009 | 0.0655 | No |
| 56 | ICA1 |  |  | 5053 | -0.012 | 0.0505 | No |
| 57 | SEC31A |  |  | 5142 | -0.014 | 0.0416 | No |
| 58 | RPS6KA3 |  |  | 5318 | -0.019 | 0.0231 | No |
| 59 | SNAP23 |  |  | 5441 | -0.022 | 0.0109 | No |
| 60 | NAPG |  |  | 5449 | -0.022 | 0.0121 | No |
| 61 | RAB22A |  |  | 5625 | -0.028 | -0.0057 | No |
| 62 | STX7 |  |  | 5762 | -0.032 | -0.0186 | No |
| 63 | VPS4B |  |  | 6113 | -0.043 | -0.0552 | No |
| 64 | RAB14 |  |  | 6246 | -0.048 | -0.0662 | No |
| 65 | VAMP7 |  |  | 6542 | -0.058 | -0.0951 | No |
| 66 | RAB2A |  |  | 6844 | -0.073 | -0.1235 | No |
| 67 | MAPK1 |  |  | 6941 | -0.078 | -0.1277 | No |
| 68 | SNX2 |  |  | 7081 | -0.086 | -0.1362 | No |
| 69 | GOSR2 |  |  | 7420 | -0.110 | -0.1656 | No |
| 70 | BET1 |  |  | 7433 | -0.111 | -0.1572 | No |
| 71 | CAV2 |  |  | 7537 | -0.121 | -0.1585 | No |
| 72 | RAB5A |  |  | 7560 | -0.123 | -0.1502 | No |
| 73 | TPD52 |  |  | 7598 | -0.128 | -0.1433 | No |
| 74 | RAB9A |  |  | 7606 | -0.128 | -0.1328 | No |
| 75 | TSG101 |  |  | 7759 | -0.147 | -0.1375 | No |
| 76 | NAPA |  |  | 7802 | -0.152 | -0.1290 | No |
| 77 | COPE |  |  | 7871 | -0.162 | -0.1226 | No |
| 78 | VAMP3 |  |  | 7982 | -0.182 | -0.1193 | No |
| 79 | CLTA |  |  | 8040 | -0.192 | -0.1091 | No |
| 80 | AP2S1 |  |  | 8103 | -0.204 | -0.0983 | No |
| 81 | YKT6 |  |  | 8147 | -0.214 | -0.0844 | No |
| 82 | BNIP3 |  |  | 8151 | -0.215 | -0.0659 | No |
| 83 | AP3S1 |  |  | 8213 | -0.232 | -0.0526 | No |
| 84 | AP2M1 |  |  | 8381 | -0.287 | -0.0467 | No |
| 85 | ARF1 |  |  | 8459 | -0.321 | -0.0273 | No |
| 86 | GNAS |  |  | 8512 | -0.344 | -0.0031 | No |
| 87 | SOD1 |  |  | 8526 | -0.349 | 0.0260 | No |
Table: GSEA details [plain text format]

  

Fig 2: HALLMARK\_PROTEIN\_SECRETION: Random ES distribution      
 Gene set null distribution of ES for **HALLMARK\_PROTEIN\_SECRETION**
